# Supplementary material for: Blockchain-Enabled Traceability in the Rice Supply Chain: Insights from the TRACE-RICE Project
Source: Foods. 2025 Oct 30;14(21):3711. doi: 10.3390/foods14213711 (PMC12607311; doi:10.3390/foods14213711)
Supplement: Supplementary file 1 [file foods-14-03711-s001.zip › foods-3906978-supplementary.pdf]

Report on field activity records for integrated rice production

Plot identification Canal 2

Register number 1433490739001

Variety of rice Ariete

Sowing tye Terrestrial

Sowing date 15/05/2023

Harvest date 12/10/2023

Record of Plant Protection Treatments

Application date	Plant Protection Treatments						
	Plant protection products used		Type of application	Dose applied			
				Total		Per Hectare	
12/06/2023	Herbicide	NOMENI	Terrestrial	1,6	L	0,075	L/ha
12/06/2023	Adjuvant	BIOPOWER	Terrestrial	10,8	L	0,5	L/ha
12/06/2023	Herbicide	LOYANT	Terrestrial	25,9	L	1,2	L/ha
30/08/2023	Fungicide	AMISTAR TOP	Terrestrial	21,6	L	1	L/ha

TRACE-RICE with Grant nº 1934, (call 2019, section 1 Agrofood) is part of the PRIMA Programme supported under Horizon 2020, the European Union's Framework Programme for Research and Innovation

PRIMA PARTNERSHIP FOR RESEARCH AND INNOVATION IN THE MEDITERRANEAN AREA

iniav Instituto Nacional de Investigação Agrária e Veterinária, I.P.

Registration of Mechanical Operations

Application date	Fertilizer used	Dose applied				Application of nutrients								
		Total		Per Hectare		N	P	K	Ca	Mg	SO_3	Fe	Intervention date	Intervention or proceedings
													08/05/2023	Grate
													11/05/2023	Mobilize
													12/05/2023	Irrigate
													15/05/2023	Sow
10/05/2023	Nergetic 20-8-10	3246	kg	150	kg/ha	20	8	10	2	0	0	0	22/05/2023	Drain
10/07/2023	Granulated Ammonium Sulfate (Ubesol)	4328	kg	200	kg/ha	21	0	0	0	0	60	0	30/05/2023	Irrigate
													08/06/2023	Drain
													14/06/2023	Irrigate
													05/09/2023	Drain
													12/10/2023	Reap

TRACE-RICE with Grant nº 1934, (call 2019, section 1 Agrofood) is part of the PRIMA Programme supported under Horizon 2020, the European Union's Framework Programme for Research and Innovation
